# Mexican Ancestral Foods (*Theobroma cacao, Opuntia ficus indica, Persea americana* and *Phaseolus vulgaris)* Supplementation on Anthropometric, Lipid and Glycemic Control Variables in Obese Patients: A Systematic Review and Meta-Analysis

**DOI:** 10.3390/foods12061177

**Published:** 2023-03-10

**Authors:** Rebeca Escutia-Gutiérrez, Ana Sandoval-Rodríguez, Marina Galicia-Moreno, Rebeca Rosas-Campos, Mónica Almeida-López, Arturo Santos, Juan Armendáriz-Borunda

**Affiliations:** 1 Department of Molecular Biology and Genomics, Institute for Molecular Biology in Medicine and Gene Therapy, Health Sciences University Center, University of Guadalajara, Guadalajara 44340, Mexico; 2 Health Sciences University Center, University of Guadalajara, Guadalajara 44340, Mexico; 3 Tecnologico de Monterrey, School of Medicine and Health Sciences, Zapopan 45201, Mexico

**Keywords:** cocoa, nopal, avocado, common bean, Mexican foods, obesity

## Abstract

Diet containing Mexican ancestral foods such as cocoa, nopal, avocado, and common bean have been individually reported to have beneficial effects on obesity and comorbidities. Methods: A systematic review and meta-analysis on the effect of Mexican ancestral foods on the anthropometric, lipid, and glycemic control variables in obese patients was performed following PRISMA guidelines. Data were analyzed using a random-effects model. Results: We selected 4664 articles from an initial search, of which only fifteen studies satisfied the inclusion criteria. Data for 1670 participants were analyzed: 843 in the intervention group and 827 in the control group. A significant reduction in body mass index (mean difference: −0.80 (−1.31 to −0.30)) (95% confidence interval), *p* = 0.002, heterogeneity I^2^ = 92% was showed after the ingestion of cocoa, nopal, avocado, or common bean. The mean difference for body weight was −0.57 (−1.93 to 0.79), waist of circumference: −0.16 (−2.54 to −2.21), total cholesterol: −5.04 (−11.5 to 1.08), triglycerides: −10.11 (−27.87 to 7.64), fasting glucose: −0.81 (−5.81 to 4.19), and insulin: −0.15 (−0.80 to 0.50). Mexican ancestral food supplementation seems to improve anthropometric, lipid, and glycemic control variables in obesity; however, more randomized controlled trials are needed to have further decisive evidence about dosage and method of supplementation and to increase the sample size.

## 1. Introduction

Food systems around the world have undergone significant changes in recent years as a result of advances in food technology, which have resulted in the increased availability, affordability, and commercialization of highly processed foods. Food structure, nutritional content, and taste have all been altered as processing methods have become more sophisticated [1,2]. In particular, the consumption of processed foods is linked to the emergence of one of the world’s most serious public health issues: obesity, the 21st-century pandemic [3]. According to the World Health Organization (WHO), there are two main factors that can lead to obesity: on the one hand, a rise in the consumption of foods that are high in fat and sugar but low in vitamins and minerals, and on the other hand, a decline in physical activity brought on by an increase in sedentary jobs, urbanization, and motorized transportation (WHO, 2021) [4]. Obesity is a chronic, low-grade inflammatory illness with multiple underlying causes. Obese individuals actually run a higher danger of contracting conditions including metabolic syndrome, arterial hypertension, dyslipidemia, type 2 diabetes mellitus (T2DM), coronary heart disease, and cerebral vasculopathy [5]. Depending on the severity of the disease and comorbidities, obesity is related to a shorter life expectancy. There are currently no efficient pharmaceutical treatments for obesity [6]. The imbalance between calorie intake and expenditure is the essential cause of obesity, an abnormal and excessive accumulation of fat that can be unfavorable to health. Similarly, it is estimated that two billion adults worldwide are overweight (WHO 2021) [7]. In agreement, the National Health and Nutrition Survey (ENSANUT 2018) indicates that the prevalence of obesity in Mexico is alarming, at 75.2% of adults aged 20 years and older; moreover, 22.2% of children aged 0 to 4 years are at risk of being overweight and 35.6% of children aged 5 to 11 years show signs of this condition [8,9].

Mexico is a country of great biodiversity, in which the essence of traditional Mexican food is based on its pre-Hispanic origins where mostly plant-based, basic foods predominate. Mexican ancestral foods are those used by the Indigenous people in pre-Hispanic periods, which contain high amounts of diverse functional nutrients such as minerals, vitamins, antioxidants, and prebiotics, among others [10]. Foods from the Mexican diet such as cocoa, nopal, avocado, and common bean have been individually studied using basic and clinical models of obesity and metabolic syndrome, demonstrating beneficial effects to health [11]. Cocoa (*Theobroma cacao*), the raw material for chocolate, belongs to the Magnoliopsida class, genus *Theobroma*, and species *Cacao*. Because of the value and importance of its seeds, it is the main fruit of this genus that is commercially cultivated. The cocoa tree is indigenous to tropical America and can be found growing naturally from Peru to Mexico [12]. Due to the high level of polyphenols such as flavonoids, epicatechin, catechin, and procyanidins in cocoa and products derived from cocoa, these foods constitute one of the key natural sources of antioxidants in Latin American diets. Cocoa contains the greatest known concentration of flavonoids, even more than green tea and red wine [13]. The many health advantages of cocoa consumption include glycemic control, cardioprotection, anticancer, anti-inflammatory, and antioxidant properties [13,14]. Nopal *(Opuntia ficus-indica)*, also identified as prickly pear cactus, is extensively spread, primarily in Mexico, Latin America, South Africa, and the Mediterranean region. Nopal has been used in traditional medicine because of its pharmacological properties, which include its capacity to act as an antiulcerogenic, antioxidant, antidiarrheal, anti-inflammatory, hypoglycemic, neuroprotective, and antihypercholesterolemic agent [15]. Several distinct nopal compounds, including ascorbic acid, vitamin E, carotenes, dietary fiber, amino acids, and antioxidant compounds, are responsible for some of these benefits (phenols, flavonoids, beta-xanthin, and betacyanin) [16]. *Persea americana* (also known as avocado, avocado pear, or alligator pear) is a flowering plant native to Mexico and Central America that belongs to the Lauraceae family. Mexico is the world’s leading avocado producer [17]. Avocadoes have a high nutritional value, as they are rich in vitamins, minerals, fiber, and phytochemicals. Avocado consumption has been associated with health benefits such as lower risk of metabolic syndrome, cardiovascular disease, and being overweight or obese due to increased satiety and decreased appetite. [18,19]. Common bean (*Phaseolus vulgaris*) is an important crop in the diet of the majority of the Mexican population. It is the third most cultivated legume in the world, after soybeans and peanuts, and Mexico is the world’s fourth largest producer [20,21]. Beans contain a high protein percentage (16–33%), as well as high concentrations of aromatic amino acids (lysine, leucine, isoleucine, aspartic acid, and glutamic acid), phenols, anthocyanins, tannins, flavonoids, lectins, phytic acid, nondigestible polysaccharides, saponins, and phytosterols. One of the most researched properties of beans is their ability to lower cholesterol [22]. Some clinical studies showed a beneficial effect of bean consumption on the glycemic index and a protective role against the establishment of T2DM; this effect seems to be due to its high content of polyphenols that confer an important antioxidant effect [23]. In addition, bean consumption has been suggested to decrease the risk of ischemic heart and cardiovascular diseases and stomach and prostate cancer, and is associated with weight and obesity control in elderly populations [24]. Unfortunately, traditional diets that include whole foods and predominately home cooking and food preparation are being replaced by processed food diets. Several studies suggest that eating processed foods may increase the risk of obesity, as well as the prevalence of metabolic syndrome, and increase total and low density lipoprotein cholesterol and the risk of hypertension [25]. The aim of this study was to perform a systematic review and meta-analysis of the beneficial effects of Mexican ancestral food on the treatment of obesity, showing clinical evidence of their ability to modulate anthropometric and biochemical parameters in patients.

## 2. Materials and Methods

### 2.1. Search Strategy

Two researchers conducted an independent systematical search to identify studies published in online medical databases from 2012 to 2022, including PubMed, Web of Science, Scopus, and the Cochrane Central Register of Controlled Trials (CENTRAL). The review process was documented using Preferred Reporting Items for Systematic Reviews and Meta-Analysis guidelines (PRISMA). The Supplementary Materials section contains the complete search strategy. As a search strategy, medical subject headings (MESH) and non-MESH terms including these keywords were used: “Cacao” OR “Cocoa” OR “Dark Chocolate” OR “Theobroma cacao” OR “Nopal” OR “Opuntia ficus indica” OR “Prickly pear cactus” OR “Common bean” OR “Phaseolus vulgaris” OR “Avocado” OR “Persea americana” AND “Obesity” OR “Weight Loss” OR “weight reduce” OR “weight decrease” OR “obese” OR “central obesity” OR “overweight” OR “adipose tissue” OR “fat mass” OR “adiposity” OR “body fat mass” OR “body fat mass (BMI)” OR “waist circumference”. “Cacao” OR “Cocoa” OR “Dark Chocolate” OR “Theobroma cacao” OR “Nopal” OR “Opuntia ficus indica” OR “Prickly pear cactus” OR “Common bean” OR “Phaseolus vulgaris” OR “Avocado” OR “Persea americana” AND “Blood Sugar” OR “Blood glucose” OR “fasting plasma glucose” OR “impaired fasting glucose” OR “Insulin Resistance” OR “HOMA IR” OR “homeostasis model assessment” OR “oral glucose tolerance test” OR “glucose tolerance test” OR “glucose intolerance” OR “Blood cholesterol” OR “Cholesterol esters” OR “Hypercholesterolemia” OR “Blood triglycerides” OR “Hypertriglyceridemic waist” OR “Triacylglycerol” OR “LDL-cholesterol” OR “Low density lipoprotein” OR “HDL-cholesterol” OR “High density lipoprotein cholesterol”. To avoid omitting any relevant articles, all reference lists of eligible articles were hand-searched. Furthermore, this study excluded unpublished articles and grey literature such as conference papers, theses, and patents.

### 2.2. Study Selection, Inclusion and Exclusion Criteria

We evaluated all clinical trials that assessed the effects of cacao, nopal, avocado, or common bean intake on anthropometric measurements such as body mass index and waist circumference; additionally, biochemical parameters such as total cholesterol, triglycerides (TAG), high-density lipoprotein-cholesterol (HDL-c), low-density lipoprotein-cholesterol (LDL-c), and glucose as well as homeostasis model assessment-estimated insulin resistance (HOMA-IR) were evaluated. Studies were included if they had the following criteria: (1) the study design was prospective, randomized, controlled, open or blinded trials, with an either parallel or crossover design, enrolling patients with overweight or obesity or patients with overweight or obesity and diabetes mellitus or metabolic syndrome; (2) patients prescribed cacao, nopal, avocado, or common bean consumption; (3) studies reported information about at least one of the following outcomes: body mass index, waist circumference, total cholesterol, triglycerides, HDL-c, LDL-c, and glucose, as well as insulin levels and HOMA-IR at baseline and at the end of the intervention; (4) studies that were done on adult subjects (>18 years); and 5) studies that were published in the English language (Table 1).

Studies were excluded if they had the following characteristics: (1) done on children, animals, or in vitro; (2) investigated the effect of other interventions along with cacao, nopal, avocado, or common bean; and (3) those that did not report body weight, body mass index, waist circumference, total cholesterol, TAG, HDL-c and LDL-c, glucose, insulin levels, or HOMA-IR at baseline and at the end of the intervention.

The outcomes were calculated as the mean value between the baseline and final levels (with the associated dispersion measures) for TAG (in mmol/L (to convert to mg/dL, divide by 0.0113)), HDL-c and LDL-c (in mmol/L (to convert to mg/dL, divide by 0.0259)), and fasting blood glucose (in mmol/L (to convert to mg/dL, divide by 0.0555). TAG (in mmol/L (to convert to mg/dL, divide by 0.0113)), HDL-c and LDL-c (in mmol/L (to convert to mg/dL, divide by 0.0259), and fasting blood glucose (in mmol/L (to convert to mg/dL, divide by 0.0555) were calculated as the mean value among the baseline and final levels (with the associated dispersion measures).

### 2.3. Meta-Analysis Determination

For the statistical analysis, Review Manager (Revman) Version 5.3 (Cochrane, London, UK) software was used. The data are presented as mean ± standard deviation and the confidence interval is 95% (CI). Heterogeneity among the studies was tested using the Cochran’s Q test, and inconsistency was tested using the I^2^ test. Study weights were assigned using the inverse variance method [26], and calculated by the random-effects model [27]. A value of *p* < 0.05 was considered statistically significant.

### 2.4. Risk of Bias Assessment

Two authors (REG and RRC) independently assessed the risk of bias of the included studies using Cochrane’s “Risk of bias” tool, which is described in Chapter 8 of the Cochrane Handbook for Systematic Reviews of Interventions [28]. Random sequence generation, allocation concealment, blinding of participants and personnel, blinding of outcome assessment, incomplete outcome data, selective reporting, and any other source of bias were all considered. In the evaluated studies, the Cochrane’s risk of bias tool determined a low, high, or unclear risk of bias. Disagreements were resolved through discussion among the review team members.

### 2.5. Assessment of Heterogeneity

Forest plots were examined visually to determine the direction and magnitude of effects, as well as the degree of overlap between confidence intervals. The I^2^ statistic was used in each analysis to measure trial heterogeneity, but we acknowledge that there is substantial uncertainty in I^2^ value when only a small number of studies were analyzed. In this case, the *p* value from the Chi-square test (X_2_) was also taken into account. Interpretation of the I^2^ statistic value was made according to Section 10.10.2 of the Cochrane Handbook for Systematic Reviews of Interventions using these ranges: 0% to 40% indicates heterogeneity might not be important, 30% to 60% may represent moderate heterogeneity, 50% to 90% may represent substantial heterogeneity, and 75% to 100% indicates considerable heterogeneity [28].

## 3. Results of the Systematic Review

A total of 4664 articles were selected from an initial search (625 from PubMed, 34 from CENTRAL, 2255 from Web of Science, and 1750 from Scopus); 2017 articles were excluded before screening due to duplication. The remaining 2647 records were screened by title and abstract, and 2491 were excluded. The full text of the remaining 118 articles was retrieved and the last decision on the inclusion of the articles was made based on the PICOS criteria. Finally, we excluded 103 articles due to a mixture of an ancestral food with another supplement in the same intervention or a lack of numerical results or a lack of baseline and final intervention data.

**Figure 1:**
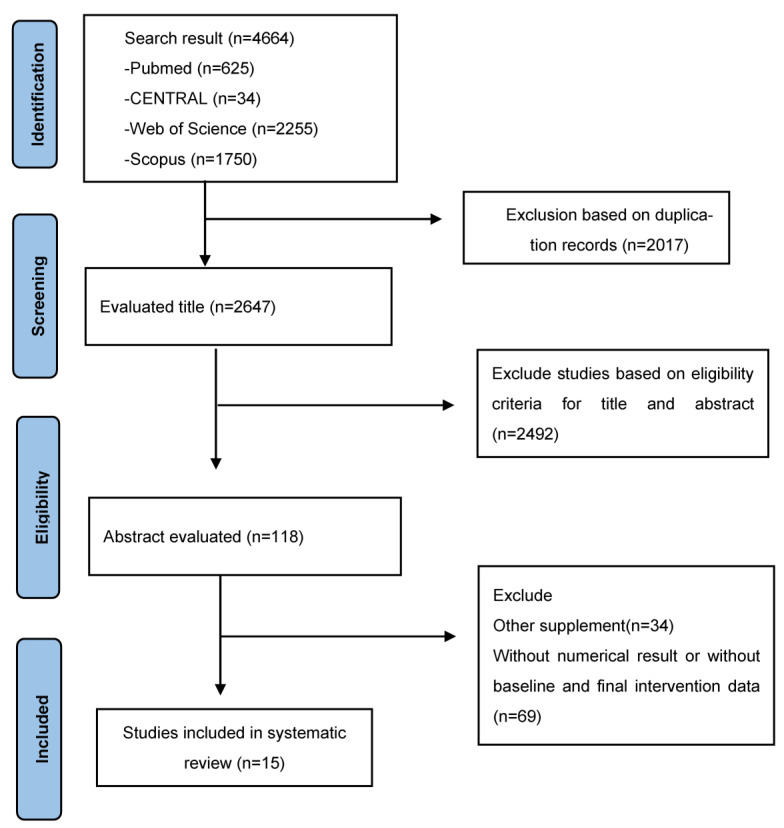
The PRISMA flow diagram depicts the process of selecting studies for systematic review and meta-analysis.

Fifteen studies satisfied the inclusion criteria and were included in the systematic review and meta-analysis (Figure 1). Eight studies assessed the effect of dark chocolate or cocoa supplementation in overweight/obese patients [29,30,31,32,33,34,35,36]. Two studies analyzed the outcome of common bean or *Phaseolus vulgaris* consumption in the condition of T2DM and obesity [37,38]. Three studies evaluated the effect of avocado ingestion on abdominal adiposity and visceral adiposity in subjects with syndrome metabolic disease and obesity [39,40,41]. Finally, two studies measured the effects of nopal or *Opuntia Ficus Indica* intake on anthropometric and metabolic characteristics in obese type 2 diabetes patients [42,43].

Patient anthropometric outcomes and serum lipid parameters enlisted in included studies are shown in detail in Table 2. Two out of the fifteen studies were double-blinded, randomized, placebo-controlled trials with parallel groups [29,30]; two studies were randomized crossover trials [37,43]; two studies had a randomized, placebo-controlled, double-blind design [35,38]; one study had a multicenter, randomized, controlled parallel-arm trial design [40]; one study was a single-center, randomized, 2-arm, controlled, parallel trial [41]; three studies were randomized controlled trials [31,34,39]; one study was a randomized, placebo-controlled, cross-over [36]; one study was a double-blind, placebo-controlled, clinical pilot trial [32]; one study was a prospective dietary intervention [42]; and finally, one study was a two-phase, randomized, double-blind, clinical trial [33]. Most of them included adults of both genders, while one study included exclusively women. The duration of the studies was variable, ranging from 4 weeks to 6 months. Eleven manuscripts were conducted on overweight/obese patients [30,31,33,34,35,36,37,38,42,43], two on T2DM and obese patients [29,41], and two on metabolic syndrome- and obesity-diagnosed patients [32,40].

Manuscripts regarding dark chocolate reported intake between 2 g to 37 g per day. Cacao flavonoid doses ranged from 80 mg/day to 270 mg/day. Method of supplementation was different in each study. In one study, participants consumed 236 mL of a sugar-free natural cocoa beverage (272 kJ/day) daily; in another protocol, participants consumed 1.45 oz of a dark chocolate tasting square, while in another study, the participants consumed 37 g/d of dark chocolate from a snack bar and a sugar-free cocoa beverage (total dose of natural cocoa: 22 g/d, total flavanols: 814 mg/day) [30,31,32,33,35,38]. Regarding cocoa supplementation, commercially available cacao bean extract powder was dissolved in water, while other studies provided encapsulated cocoa powder to subjects [29,34]. Dark chocolate or cocoa significantly diminished total cholesterol and triglyceride levels and improved glycemic control. Biochemical outcomes are shown in detail in Table 2.

The method of supplementation for *Phaseolus vulgaris* in the analyzed studies was diverse: one study recommended two capsules three times a day (t.i.d.) (400 mg per capsule) before meals, for a total of 2400 mg per day. In another study, the daily intake of common bean was in a 32 g-baked snack bar. *Phaseolus vulgaris* supplementation showed changes in anthropometric parameters, leading to a significant reduction in body mass index, waist circumference, and body fat [37,38]. Hass avocado consumption in the studies was one raw piece/day (approximately 200–300 g) included in a meal. A significant reduction in total and LDL-c was observed, and a change in distribution of abdominal adiposity in obese subjects [40]. Nopal ingestion in the studies varied from 6.2 g to 300 g per day. Supplementation method included fresh nopal in sterile plastic bags every week. Every week, fresh nopal was placed in sterile plastic bags as a supplement. According to one study, 300 g (2 cups) of boiled nopal (*Opuntia ficus-indica*) obtained from 375 g of fresh nopal cladodes (100 g = 16 kcal and 2.2 g of dietary fiber) corresponds to 3% of total energy intake and 33% of the daily recommendation for dietary fiber (25 g) on a 2000 kcal diet [37]. In another study, participants consumed 2 cups (280 g) of nopales per day. Nopales were cooked in boiling water for ten minutes, placed in ice to cool, packed in 140 g bags (equivalent to 1 cup), and refrigerated before distribution. Daily nopal consumption significantly decreased weight, BMI, waist, and hip circumference [43].

### 3.1. Risk of Bias Assessment

**Figure 2:**
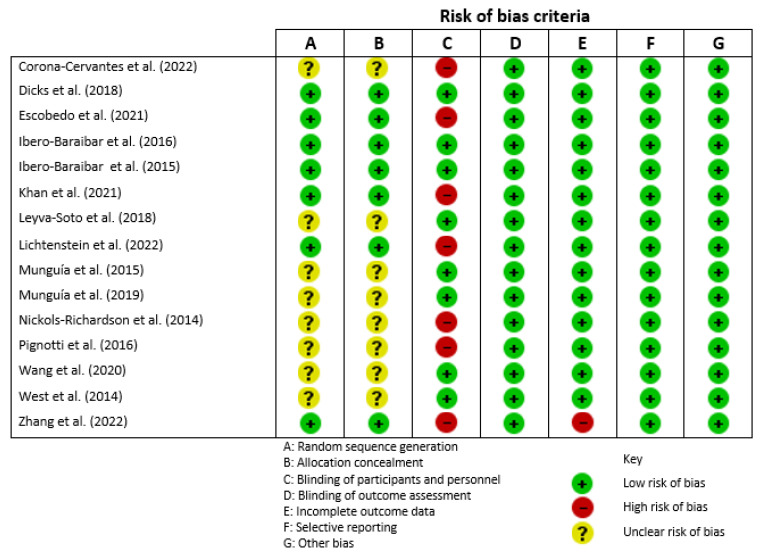
Assessment of the risk of bias in the included studies according to the Cochrane criteria [29,30,31,32,33,34,35,36,37,38,39,40,41,42,43].

As shown in Figure 2, the majority of randomized controlled trials (53.3%) had an unclear risk of bias for A and B criteria, random sequence generation, and allocation concealment due to insufficient information about the sequence generation process to allow the decision of “Low risk” or “High risk” [32,33,34,35,36,38,42,43]. Moreover, 46.6% of studies had a low risk in A and B criteria [29,30,31,37,39,40,41]. In the mentioned studies, researchers define a random component in the sequence generation process using the “rand function” random number between 1 and 2 in Microsoft Office Excel [30,31]. Furthermore, some studies used permuted block randomization [29,40]; in others, randomization sequences for the participants were computer-generated [37], while a study randomized participants to a code (blinded allocation), based on a blinded randomization schedule, and these codes were used on all documentation and tube labeling [41]. Concealment of allocation was maintained using sequentially numbered containers for meal dispensation [39], and finally the allocation was concealed using an interactive web response system for participant mix up [40]. Concerning the C criteria, blinding of participants and personnel was the only criterion that generated a high risk of bias in 40% of the studies [34,37,39,40,41,42,43]. Specifically, some Mexican ancestral foods did not have an ideal placebo that could achieve successful blinding of the participants, like common bean, avocado, and nopal [37,40,43]. This situation occurred when the piece or pieces of Mexican ancestral food were delivered to participants fresh or unprocessed. However, the majority of the studies (60%) had low risk of bias in this criterion, fulfilling an important requirement for a clinical trial [29,30,31,32,33,35,36,38]. All studies had a low risk of bias in the remaining criteria, such as blinding of outcome assessment (D criteria), selective reporting (F criteria), and others bias (G criteria). All studies showed complete data of outcomes evaluated, described the methodology used to determine anthropometric, lipid, biochemical, and glycemic parameters, and enlisted dropouts [29,30,31,32,33,34,35,36,37,38,39,40,41,42,43]. Regarding the incomplete outcome data (E criteria), one study had a high risk of bias because the dropout rate was 21.8%, which is a usual percentage in a short intervention study, but it could be considered a limitation when compared with a per-protocol with intent-to-treat analysis [41]. The remaining studies had low risk of bias due to no missing outcome data.

### 3.2. Mexican Ancestral Foods Improve Some Anthropometric Measurements

Obesity is a worldwide epidemic and a major risk factor for many of the most common diseases. Anthropometric measures are simple, low-cost, non-invasive tools for detecting obesity and assessing the risk of morbidity and mortality [44]. Body mass index (BMI), waist circumference (WC), waist-to-hip (WHR) and waist-to-height ratios, visceral fat area (VFA), body fat percentage (BFP), and body shape index (ABSI) are the most commonly used [45]. Body mass index (BMI) is defined as weight in kilograms divided by height in square meters and is the most widely used index for quantifying obesity (kg/m^2^). In agreement with the WHO, obesity is defined as a BMI ≥ 30 kg/m^2^ [7]. BMI has been one of the most widely used weight-related anthropometric measures. However, the disease expectedness of BMI is restricted, as it does not differentiate between muscle and fat accumulation or distribution of adipose tissue. As a result, BMI does not interpret altered phenotypes of obesity and the relation of fat distribution or alteration between subcutaneous and visceral adiposity [46]. Primarily, central visceral adiposity is a strong predictor for metabolic risk factors. Waist circumference (WC) is a simple anthropometric parameter to measure abdominal adiposity in clinical practice. WC is powerfully associated with cardiovascular mortality. Consequently, it has been suggested, in accordance with a 2008 expert consultation report of the WHO, that WC in combination with BMI are the best tools to assess metabolic risk [47].

A study by Nickols-Richardson et al. (2014) investigated the effect of incorporating a sugar-free natural cocoa beverage and extra dark chocolate compared with a combination of sugar free cocoa-free vanilla beverage and non-chocolate sweet snack. During the study, subjects in both groups followed an energy-restricted diet (ERD) with a macronutrient composition of 50% carbohydrate, 30% fat, and 20% protein planned to provoke approximately 0.91 kg per week of body weight loss by consuming 2092 fewer kJ per day than theorical energy requirements. This study revealed reductions in anthropometric parameters: women in the dark chocolate group lost 5.3% of body weight, while women in the non-chocolate group were missing 5.9% of body weight from baseline to intervention-end at week 18. BMI, waist and hip circumference, and body fat percentage all decreased after 18 weeks of supplementation in both groups at any interval and over time. The study’s limitation was that it did not include a control group that did not consume a snack or beverage, or follow ERD, so more research is needed [34].

A double-blind, placebo-controlled clinical pilot trial in overweight subjects who met metabolic syndrome criteria yielded similar results. Munguía et al. (2015) investigated the effects of daily consumption of foods supplemented with cocoa extract on a hypocaloric diet in obese adults, with the control group receiving only hypocaloric meals. Following 4 weeks of intervention, body weight loss was greater in the cacao supplement (2.4 kg) group compared with the placebo (1.7 kg) group (3.0 vs. 2.1%). Abdominal circumference was also significantly reduced in cacao (3.5 cm) versus placebo (1.8 cm), (3.6 vs. 1.8%, respectively) [32].

On the other hand, a randomized controlled clinical trial, including a total of 105 adults with a BMI of less than 25 kg/m2, were divided into two groups: one had a daily meal that included one fresh Hass avocado, and the other received an isocaloric meal with the same ingredients but no avocado for 12 weeks. Among women, the treatment group showed a greater reduction in change of visceral adipose tissue (ΔVAT), control (1.6 ± 89.8 g) compared with treatment (−32.9 ± 81.6 g). According to sex, there were significant differences in adiposity factors, with women showing a higher reduction in SAAT (−61.2 ± 152.7 g, control) compared with the treatment group (13.7 ± 133.1 g), while the control male group had a higher ratio of visceral to subcutaneous abdominal adipose tissue (VS Ratio) (0.43 ± 0.14 vs. 0.30 ± 0.09). Among men, in terms of changes in abdominal adiposity or glycemic outcomes, there were no significant differences between groups [39]. A randomized, double-blind, placebo-controlled study on obese adults was conducted to assess weight loss due to regular intake of *Phaseolus vulgaris* extract (PVE). Wang et al. (2020) discovered that after 35 days, the average weight loss in the PVE group was 2.24 kg versus 0.29 kg in the placebo group. The intervention group received two capsules before each of three daily meals, for a total of 2400 mg per day over 35 days. In the control group, a placebo containing maltodextrin (1632 kJ/100 g) was used. The body mass index decreased by an average of 0.79, and body fat decreased by 1.53% on average compared with baseline quantifications in PVE-supplemented subjects. Additionally, BMI decreased by 0.79 kg/m^2^ in the PVE group and 0.1 kg/m^2^ in the placebo group [38].

A study of Dicks et al. (2018) evaluated whether regular ingestion of flavanol-rich cocoa powder might improve lipid metabolism in subjects with T2DM and overweight through a double-blinded, randomized, placebo-controlled trial with parallel group design. Participants were allocated to two groups: the first group received five capsules of cocoa power daily (0.5 g per capsule, for a total of 2.5 g/day of a flavanol-rich cocoa) for 12 weeks, and the second group received five capsules of 0.5 g pure microcrystalline cellulose. Nontransparent capsules of hydroxypropylmethyl cellulose with an equal appearance were chosen for both groups because they disintegrate and dissolve in the upper gastrointestinal tract. Dicks et al. discovered that the cocoa group significantly reduced waist circumference (103.6 ± 4.8 cm vs. 102.3 ± 4.6 cm, *p* = 0.047) and waist-to-hip ratio (0.97 ± 0.02 vs. 0.96 ± 0.02, *p* = 0.011). However, the cocoa treatment had no effect on serum triglyceride, total cholesterol, LDL-c, or HDL-c concentrations [29].

### 3.3. Mexican Ancestral Foods Effect on Lipid Biochemical Profile

Changes in lipid metabolism are frequently observed in obese patients. Obese patients are dyslipidemic in 60–70% of cases. Obese patients’ lipid abnormalities include elevated serum triglyceride, very low density lipoprotein (VLDL), apolipoprotein B, and non-HDL-c levels [48]. Corona-Cervantes et al. (2022) conducted a study to assess the ability of nopal to improve the health of obese participants through a physical and dietary intervention. They identified that blood levels of glucose (112 ± 41 vs. 98 ±, 26 mg/dL), total cholesterol (190 ± 34 vs. 178 ± 23 mg/dL), and HDL-c (41.3 ± 9.9 vs. 39.2 ± 9.0 mg/dL) decreased significantly in obese women that consumed 2100 g of nopal per week for 30 days and were given a customized diet plan with a 500 kcal daily caloric restriction as opposed to the control group. This group received the same nopal portions of the obese group but did not obtain a customized diet plan with energy restrictions during the study and continued their normal lifestyle. This could be a consequence of the multiple bioactive compounds of nopal, such as flavonoids, fibers and vitamins, that contribute to changes in specific biochemical parameters [42]. A randomized clinical trial was conducted in 20 overweight participants with impaired blood lipids; participants consumed either 32 g of a baked common bean snack or non-baked bean snack (control) for four weeks. Consumption of the common bean reduced apolipoprotein B-100 levels (74.2 ± 26.4 vs. 56.6 ± 12.7 mg/dL). This decrease can be attributed to the increased consumption of dietary fiber while eating common beans. The common bean baked snack could be consumed without endangering cardiovascular health even though it did not significantly alter any other lipid or blood glucose indicators [37].

West et al. (2013) described a randomized placebo-controlled, cross-over study with a cocoa treatment period in which participants consumed 37 g/d of dark chocolate and a sugar-free cocoa beverage (total cocoa of 22 g/d, 814 mg/d of total flavanols). A low-flavanol chocolate bar and a cocoa-free beverage mix with no added sugar (3 mg/d total flavanols) were used as the control treatment. Among the fasting blood variables, only insulin concentration (11.8 ± 1.1 vs. 9.5 ± 1.1 µU/mL) and HOMA-IR (2.7 ± 0.27 vs. 2.23 ± 0.27) showed significant increase after control treatment compared with the dark chocolate treatment [36]. Munguía Levy et al. (2019) conducted a four-week, two-phase, randomized, double-blind clinical trial to see if a cocoa supplement high in flavonoids could improve plasma markers of oxidative stress and inflammation and physical performance in overweight middle-aged and older patients. Subjects were randomly assigned to one of three groups: placebo, highly alkalinized (no-flavonoid) supplement, or flavonoid-rich natural cocoa beverage. Total serum cholesterol levels showed a significant reduction in the placebo group (11.4 ± 19.6 md/dL). LDL-c levels decreased in both groups, but with a larger decrease (11.5 ± 18.6 mg/dL) in the flavonoids group. HDL cholesterol levels increased significantly (3.2 ± 4.3 mg/dL) only in the flavonoids group. Triglyceride levels had a significant decrease of 23.6 ± 38 mg/dL in the flavonoids group [33].

A multicenter, randomized, controlled parallel-arm trial (HAT, Habitual Diet and Avocado Trial) was designed to see if eating one large avocado per day for six months would reduce visceral adiposity in a diverse group of free-living subjects with obesity and an elevated waist circumference compared with a habitual diet. Lichtenstein et al. discovered significant reductions in total cholesterol (19,039 vs. 18,540 mg/dL) and LDL-c (11,434 vs. 11,034 mg/dL) in adults who consumed avocado supplementation versus the control group (habitual diet without avocado). Modifications in body weight, body mass index, insulin, and VLDL-c concentrations were similar between the two groups [40]. A study by Pignotti et al. (2016) evaluated the effect of nopales (prickly pear cactus pads) to improve cardiometabolic risk factors and oxidative stress in obese, hypercholesterolemic adults. In a randomized crossover trial, participants were given 2 cups/day of cucumbers (control) for two weeks; after a 2 to 3 week washout period, participants underwent a two-week supplementation period of nopal in the same amounts. For any dietary composition data, lipid profile, cardiometabolic outcomes, or oxidative stress markers, there was no significant treatment-by-time effect. When compared with baseline, both treatments significantly increased triglyceride concentrations (control 150.2 ± 87.4 vs. 172.5 ± 108.3 and nopal 164.6 ± 124.4 vs. 189.7 ± 117.1). On average, LDL-c decreased after the nopal phase (135.9 ± 21.5 vs. 132.0 ± 20.1) [43].

### 3.4. Changes in Glucose, Insulin and HOMA-IR after Mexican Ancestral Foods Supplementation

HOMA-IR is a homeostatic model assessment (HOMA) tool used to assess insulin resistance (IR) and beta-cell function. The proportion of fasting plasma glucose and insulin levels is used to calculate HOMA-IR [49]. Leyva et al. (2018) investigated the effect of flavonoid-rich chocolate consumption on the improvement of biochemical parameters associated with cardiovascular risk and metabolic syndrome in young Mexican adults. Both HOMA-IR (2.4 ± 1.5 vs. 1.93 1.1) and fasting plasma glucose (111.67 ± 10.9 vs. 91.23 ± 9.25 mg/dL) were significantly reduced after 6 months of daily consumption of the flavonoid-rich chocolate [35].

**Figure 3:**
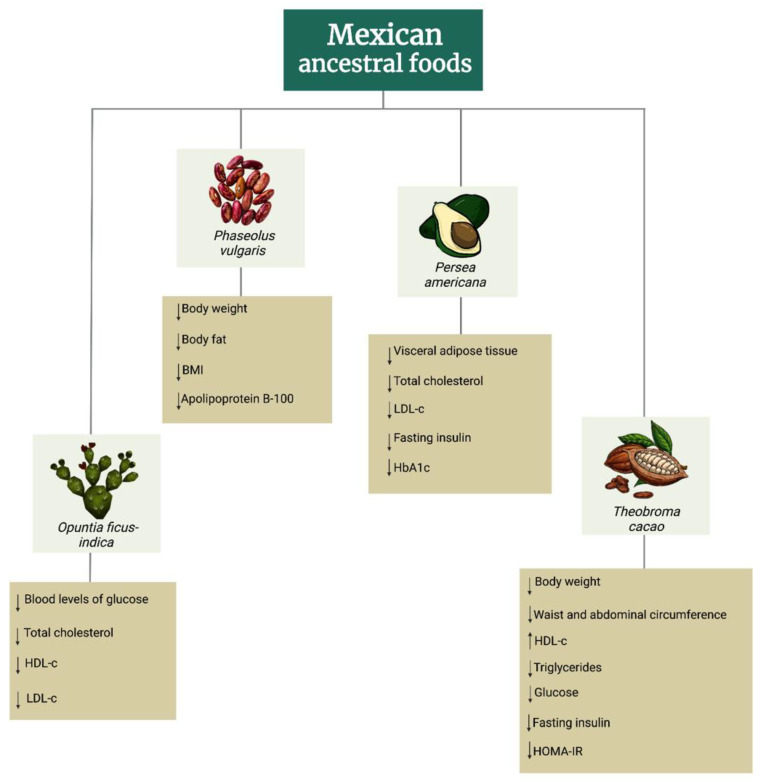
Effects of Mexican ancestral foods supplementation on obese patients [29,30,32,35,37,38,39,40,41,42,43]. BMI, body mass index; LDL-c, low density lipoprotein cholesterol; HDL-c, high density lipoprotein cholesterol; HbA1c, hemoglobin A1c; HOMA-IR, homeostatic model assessment for insulin resistance. ↓ diminution in control variables; ↑ increase in control variables.

Nickols Richardson et al. (2014) performed a randomized clinical trial with overweight/obese premenopausal women who followed an 18-week energy-restricted diet that included two sweet snacks of dark-chocolate or non-chocolate snacks plus a sugar-free drink once a day; the results showed significant reduction in glucose (4.86 ± 0.13 vs. 4.61 ± 0.12 mmol/L) and insulin concentrations (36.8 ± 4.06 vs. 36.11 ± 4.56 pmol/L) [34]. Ibero-Baraibar et al. (2015) designed a double-blind, randomized, placebo-controlled parallel nutritional intervention. They investigated the effect of cocoa extract supplementation on the overall nutritional, cardiometabolic, and oxidative grade of middle-aged overweight or obese subjects. A run-in period was carried out 1 week before the beginning of the intervention. During that period, 50 subjects were fortified to exclude cocoa and cocoa-containing products from their habitual diet. After the run-in period, 25 participants consumed 15% energy-restricted diets and were randomly allocated to obtain ready-to-eat meals supplemented with 1.4 g cocoa extract/day (645 mg total polyphenols/day), while 25 subjects consumed the 15% energy-restricted diets and received the same meals without cocoa supplementation for 4 weeks. An improvement was showed in the HOMA-IR of the cocoa group compared with the control group after 4 weeks (1.3 ± 1.0 vs. 1.2 ± 1.2), as well as the insulin levels (5.4 ± 4.2 vs. 4.7 ± 4.4 µU/mL) [30]. A randomized, placebo-controlled, two-arm parallel trial was performed by Zhang Xuhuiqun et. al. (2022) to compare the effect of carbohydrate-derived energy to avocado-derived energy on glucose homeostasis and cardiometabolic risk factors in overweight or obese subjects with insulin resistance. Participants were randomly allocated to two arms: in the first arm, subjects consumed 1 Hass avocado per day for 12 weeks (48–168 g pulp) with varied recipe suggestions. In the second arm, participants were given control foods in various combinations to match the energy level of one avocado per day as closely as possible (mini bagels, pierogis, fruit juice, waffle, instant oatmeal). Avocado supplementation showed a trend for lower fasting insulin after 12 weeks compared with control food intake (126 ± 9.42 vs. 133 ± 10.1). It is worth highlighting that glucose control was improved by lower HbA1c (5.54 ± 0.0589 vs. 5.60 ± 0.0671) after 12 weeks of avocado compared with the control food intervention [41]. A study by Ibero-Baraibar et al. (2016) analyzed the acute postprandial response after consumption of cocoa extract during the immediate 3 h of intake (postprandial 1) on blood biochemical and blood pressure markers before and after 4 weeks of its daily consumption (postprandial 2). A meal enhanced with 1.4 g of cocoa extract (415 mg flavanols) was given to one arm of overweight or obese volunteers, while the other participants were given an identical meal without the cocoa extract (control group). Both the AUC of glucose and the lipid metabolism variables during the postprandial 1 and postprandial 2 tests did not differ between the groups. When the AUC changes (post-prandial 2 vs. postprandial 1) were compared between the groups, both groups showed reduced AUC of total cholesterol and HDL-c at postprandial 2, but no statistical differences were seen [31]. Figure 3 schematizes the effects of Mexican ancestral foods on obese patients.

### 3.5. Results of the Effect of Mexican Ancestral Food on Anthropometric and Biochemical Parameters

#### Meta-Analysis

1. Body weight

**Figure 4:**
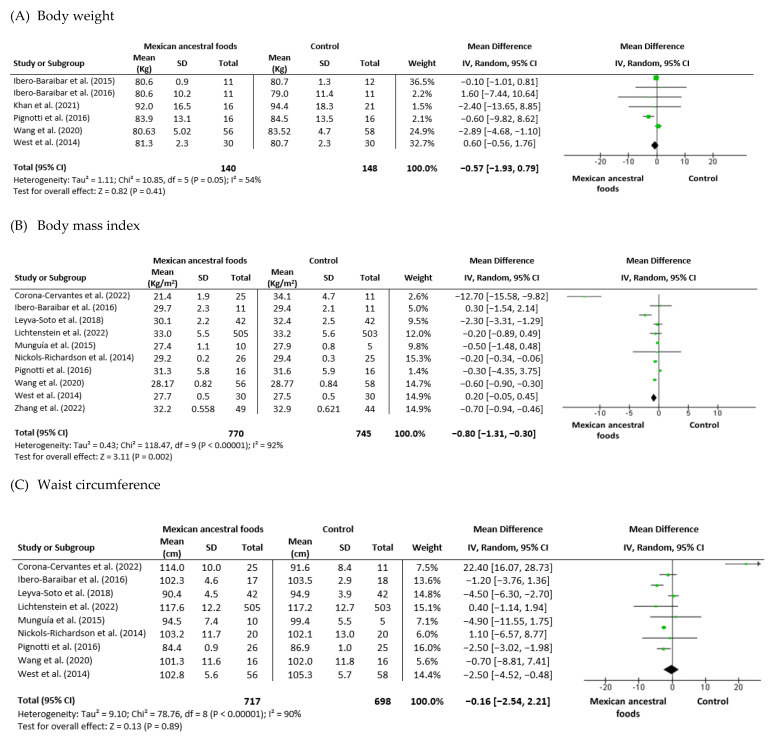
Effect of Mexican ancestral foods supplementation on (**A**) Body weight [30,31,36,38,39,43] (**B**) Body mass index [31,32,34,35,36,38,40,41,42,43] and (**C**) Waist circumference [31,32,34,35,36,38,40,42,43]. All data are presented as mean ± standard deviation and confidence interval is 95% (CI). Study weights were assigned using the inverse variance method and random-effects model. The diamond (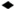) represents the overall effect estimate of the meta-analysis and the green point (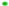) represents mean difference of effect measure of each study.

Six trials analyzed participants body weight: three trials had cocoa or dark chocolate intervention, one trial involved nopal supplementation, one study implicated avocado ingest, and one study had common bean supplementation. These studies had 140 participants in the Mexican nutraceutical intervention group and 148 in the control group. Using the inverse-variance method, heterogeneity (I_2_) was 54%(P_Heterogeneity_ = 0.05). The mean difference was −0.57 (−1.93 to 0.79) (95% CI) for the intervention versus control group (Figure 4A).

2. Body mass index

Ten trials were analyzed for body mass index: five trials had cocoa or dark chocolate intervention, two trials involved nopal supplementation, two studies implicated avocado ingestion, and one study had common bean supplementation. These studies had 770 participants in the Mexican nutraceutical intervention group and 745 in the control group. Using the inverse-variance method, a significant reduction in BMI was observed in participants who received the Mexican nutraceutical compared with the control group. Heterogeneity (I_2_) was 92%(P_Heterogeneity_ = 0.00001) and test for overall effect *p* = 0.002. The mean difference was −80 (−1.31 to −0.30) (95% CI) for the intervention versus control group (Figure 4B).

3. Waist Circumference

Nine trials include analysis of waist circumference: five trials had cocoa or dark chocolate intervention, two trials involved nopal supplementation, one study implicated avocado ingestion, and one study had common bean supplementation. These studies had 717 participants in the Mexican nutraceutical intervention group and 698 in the control group. Heterogeneity (I_2_) was 90% (P_Heterogeneity_< 0.00001). The mean difference was −0.16 (−2.54 to −2.21) (95% CI) for the intervention group versus control group (Figure 4C).

4. Total cholesterol

**Figure 5:**
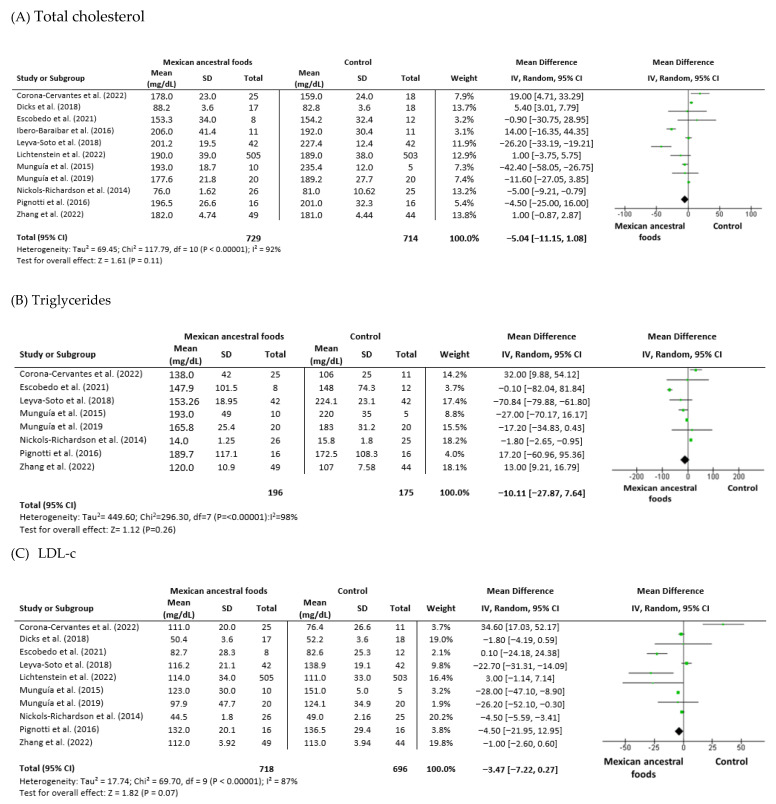

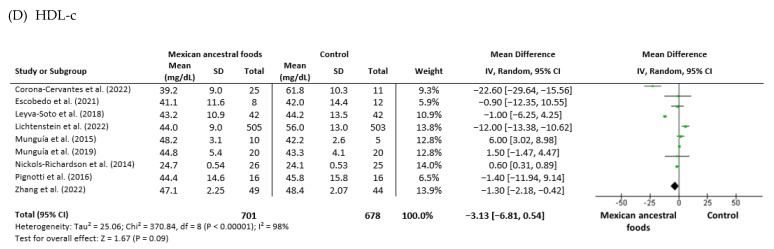
Effect of Mexican ancestral foods supplementation on (**A**) Total cholesterol [29,31,32,33,34,35,37,40,41,42,43], (**B**) Triglycerides [32,33,34,35,37,41,42,43], (**C**) LDL-c [29,32,33,34,35,37,40,41,42,43] and (**D**) HDL-c [32,33,34,35,37,40,41,42,43]. All data are presented as mean ± standard deviation and confidence interval is 95% (CI). Study weights were assigned using the inverse variance method and random-effects model. The diamond (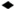) represents the overall effect estimate of the meta-analysis and the green point (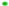) represents mean difference of effect measure of each study.

Eleven trials were analyzed for serum total cholesterol levels: six trials had cocoa or dark chocolate intervention, two trials involved nopal supplementation, two studies implicated avocado ingestion, and one study had common bean supplementation. These studies had 729 participants in the Mexican nutraceutical intervention group and 714 in the control group. Heterogeneity (I_2_) was 92% (P_Heterogeneity_ = 0.00001). The mean difference was −5.04 (−11.15 to 1.08) (95% CI) for the intervention versus control group (Figure 5A).

5. Triglycerides

Eight trials were analyzed for triglycerides: four trials had cocoa or dark chocolate intervention, two trials involved nopal supplementation, one study implicated avocado ingestion, and one study had common bean supplementation. These studies had 196 participants in the Mexican nutraceutical intervention group and 175 in the control group. Heterogeneity (I_2_) was 98% (P_Heterogeneity_ = 0.00001). The mean difference was −10.11 (−27.87 to 7.64) (95% CI) for the intervention versus control group (Figure 5B).

6. Low-density lipoprotein-cholesterol

Ten trials included analysis for LDL-c: five trials had cocoa or dark chocolate intervention, two trials involved nopal supplementation, two studies implicated avocado ingestion, and one study had common bean supplementation. These studies had 718 participants in the Mexican nutraceutical intervention group and 696 in the control group. Heterogeneity (I_2_) was 87% (P_Heterogeneity_ = 0.00001). The mean difference was −3.47 (−7.22 to 0.27) (95% CI) for the intervention versus control group (Figure 5C).

7. High-density lipoprotein-cholesterol

Nine trials were analyzed for HDL-c: four trials had cocoa or dark chocolate intervention, two trials involved nopal supplementation, two studies implicated avocado ingestion, and one study had common bean supplementation. These studies had 701 participants in the Mexican nutraceutical intervention group and 678 in the control group. Heterogeneity (I_2_) was 98% (P_Heterogeneity_ = 0.00001). The mean difference was −3.13 (−6.81 to 0.54) (95% CI) for the intervention versus control group (Figure 5D).

8. Fasting plasma glucose

**Figure 6:**
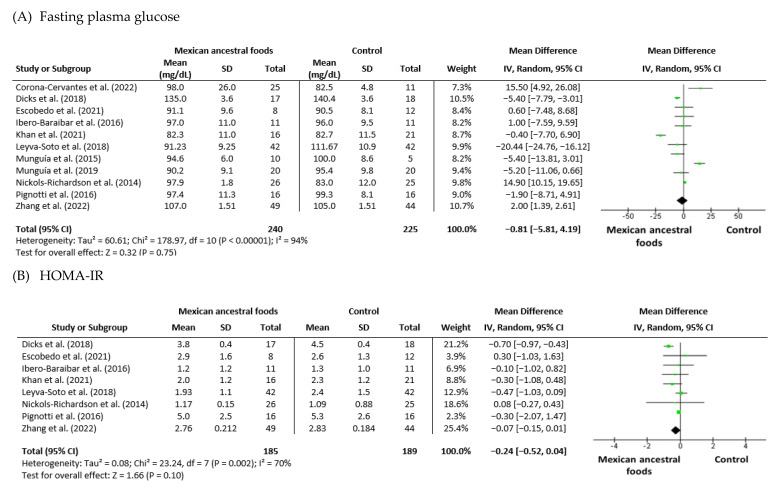

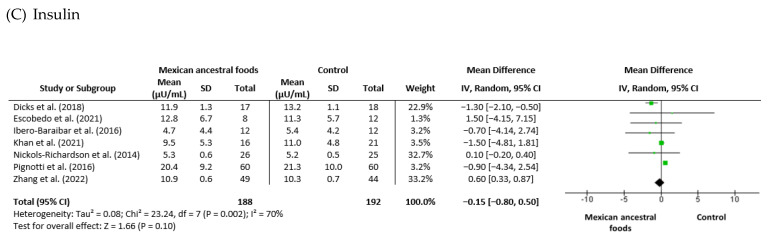
Effect of Mexican ancestral foods supplementation on (**A**) Fasting plasma glucose [29,31,32,33,34,35,37,39,41,42,43], (**B**) HOMA-IR [29,31,34,35,37,39,41,43] and (**C**) Insulin [29,31,34,37,39,41,43]. All data are presented as mean ± standard deviation and confidence interval is 95% (CI). Study weights were assigned using the inverse variance method and random-effects model. The diamond (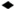) represents the overall effect estimate of the meta-analysis and the green point (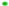) represents mean difference of effect measure of each study.

Eleven trials were analyzed for levels of glucose in fasting plasma: six trials had cocoa or dark chocolate intervention, two trials involved nopal supplementation, two studies implicated avocado ingestion, and one study had common bean supplementation. These studies had 240 participants in the Mexican nutraceutical intervention group and 225 in the control group. Heterogeneity (I_2_) was 94% (P_Heterogeneity_ = 0.00001). The mean difference was −0.81 (−5.81 to 4.19) for the intervention versus control group (Figure 6A).

9. HOMA-IR

Eight trials were analyzed for HOMA-IR index: four trials had cocoa or dark chocolate intervention, one trial involved nopal supplementation, two studies implicated avocado ingestion, and one study had common bean supplementation. These studies had 185 participants in the Mexican nutraceutical intervention group and 189 in the control group. Heterogeneity (I_2_) was 70% (P_Heterogeneity_ = 0.002). The mean difference was −0.24 (−0.52 to 0.04) (95% CI) for the intervention versus control group (Figure 6B).

10. Insulin

Seven trials included analysis for insulin: three trials had cocoa or dark chocolate intervention, one trial involved nopal supplementation, two studies implicated avocado ingestion, and one study had common bean supplementation. These studies had 188 participants in the Mexican nutraceutical intervention group and 192 in the control group. Heterogeneity (I_2_) was 75% (P_Heterogeneity_ = 0.0005). The mean difference was −0.15 (−0.80 to 0.50) (95% CI) for the intervention versus control group (Figure 6C).

## 4. Discussion

Overweight and obesity continue to rise worldwide, with the number of people with excess body weight attainment more than 2 billion, representing approximately 30% of the world population [50,51]. Obesity is a main health challenge since it increases the risk of other chronic diseases, such as type 2 diabetes mellitus and metabolic syndrome. Obesity diminishes quality of life and life expectancy [52,53]. Guidelines of the National Institute of Health (NIH) and World Health Organization (WHO) recommended BMI as a measure to classify people as obese instead of traditional height vs. weight charts. BMI overweight classification is as follows: a BMI greater than or equal to 25 to 29.9 kg/m^2^ is considered overweight and a BMI greater than or equal to 30 kg/m^2^ is considered obese [7,54]. According to this, in most of the literature, obesity has been defined using the BMI, while body adiposity and fat distribution have not been considered [45]. Mexico is a country rich in biodiversity, and the essence of the basic foods that comprise traditional Mexican cuisine from its pre-Hispanic origins has been preserved [11,25]. Food is considered traditional when it preserves cultural patterns that have been passed down from generation to generation, while new products are introduced over time, increasing nutritional diversity [10,25]. To the best of our knowledge, this is the first systematic review with a meta-analysis reporting the effects of Mexican ancestral foods on the anthropometrical, lipid, and glycemic control variables in obesity. The different outcome measures for quantitative analysis were body weight, BMI, waist circumference, total cholesterol, triglycerides, LDL-c, HDL-c, fasting plasma glucose, HOMA-IR, and insulin. We performed a meta-analysis to compare the intervention against control groups.

Body mass index was used as a primary outcome in the analyzed studies. The studies by Corona-Cervantes et al. (2022), Ibero-Baraibar et al. (2016), and Wang et al. (2020) found a significant difference in post-supplementation BMI in the intervention groups versus control groups [31,38,42]. However, the studies by Leyva-Soto et al. (2018), Lichtenstein et al. (2022), Munguía et al. (2015), Nickols-Richardson et al. (2014), Pignotti et al., West et al. (2014), and Zhang et al. (2022) could not find a significant difference in BMI [32,34,35,36,40,41,43]. Meta-analysis of BMI showed considerable heterogeneity between the studies, at I^2^ = 92%. We found a combined negative effect of BMI, where the mean value of BMI was lower in the intervention group after supplementation ((−0.80 (−1.31 to −0.30)) (95% CI). Nine studies reporting on body weight were included in the meta-analysis. Munguía et al., 2015, Nickols Richardson et al., 2014, and Wang et al., 2020 found a significant difference after the intervention, as compared with controls [32,34,38]. Six other studies, by Ibero-Baraibar et al. (2016), Ibero-Baraibar et al. (2015), Khan et al. (2021), Leyva-Soto et al. (2018), Pignotti et al. (2016), and West et al. (2014) did not find a significant difference in body weight between the intervention and control groups [30,31,35,36,39,43]. Meta-analysis of body weight indicated moderate heterogeneity among studies, at I^2^ = 56%. The mean body weight value in the intervention group was reduced ((−0.57 (−1.93 to 0.79)) (95% CI).

Analysis of waist circumference showed a positive effect of the intervention, but there was no significant difference between the intervention and control groups. Out of nine studies, only three, by Leyva-Soto et al. (2018), Munguía et al. (2015), and Wang et al. (2020) showed a significant difference in waist circumference in the intervention group [32,35,38]. The studies by Munguía et al. (2019) and Nickols-Richardson et al. (2014) found a significant decrease in post-intervention waist circumference in both the control and intervention groups; however, there was no statistical difference between them [33,34]. The lasting studies by Corona-Cervantes et al. (2022), Dicks et al. (2018), Lichtenstein et al. (2022), and Pignotti et al. (2016) found no modifications in the intervention group compared with the control group at the baseline and end of supplementation [29,40,42,43]. Meta-analysis of waist circumference revealed considerable heterogeneity (I^2^ = 90%) and also negative combined effects, which suggests that the mean value was lower in the intervention group ((−0.16 (−2.54 to 2.21)) (95% CI). Abnormal plasma lipid levels (dyslipidemia), characterized by high levels of total cholesterol, LDL-c, triglycerides, and reduced HDL-c levels, is a combination associated with obesity and the development of T2DM [48]. Thirteen studies including total cholesterol serum levels were included in this meta-analysis; one study by Ibero-Baraibar et al. (2016) found a significant reduction in this parameter in the cocoa intervention group [31]. Meta-analysis of total cholesterol levels indicated considerable heterogeneity among studies, at I^2^ = 92%. The mean of the total cholesterol value in the intervention group was reduced ((−5.04 (−11.15 to 1.08)) (95% CI). Two studies out of nine regarding triglycerides, one by Leyva-Soto et al. (2018) and the other by Munguía et al. (2015), showed a significant reduction in the intervention group compared with the control group [32,35]. Meta-analysis of triglycerides showed considerable heterogeneity among studies, at I^2^ = 98%. The mean triglycerides value in the intervention group was reduced ((−10.11 (−27.87 to 7.64)) (95% CI). Regarding LDL-c, ten studies included found no changes in the intervention group; however, the study by Leyva-Soto et al. (2018) demonstrated a significant decrease in the dark chocolate intervention subjects [35]. Meta-analysis of LDL-c indicated considerable heterogeneity among studies, at I^2^ = 87%. The mean LDL-c value in the intervention group was reduced ((−3.47 (−7.22 to 0.27)) (95% CI). Analysis of eight studies with HDL-c data showed no changes after intervention; only the study by Munguía et al. (2015) found a significant difference in cocoa supplementation compared with the control group [32]. Meta-analysis of HDL-c indicated moderate heterogeneity among studies, at I^2^ = 56%. The mean HDL-c value in the intervention group was reduced ((−3.13 (−6.81 to 0.54)) (95% CI).

Obesity and overweight are major risk factors for T2DM because body weight gain increases the risk of T2DM, especially when combined with excess body fat gain [44,55]. In this systematic review and meta-analysis, the subjects included showed comorbidities associated with obesity, and the analyses specifically included outcomes measured to diagnose T2DM. Regarding the changes in fasting plasma glucose, HOMA-IR, and insulin among the selected studies, the analysis of fasting plasma glucose showed a moderate effect of the intervention, but no significant difference between the intervention and the control groups. Two out of the eleven studies included, one by Nickols-Richardson et al. (2014) and another by Lichtenstein et al. (2022), found a significant reduction in fasting plasma glucose level in the intervention group compared with the control group [34,40]. Meta-analysis of fasting plasma glucose indicated considerable heterogeneity among studies, at I^2^ = 94%. The mean fasting plasma glucose value in the intervention group was reduced ((−0.81 (−5.81 to 4.19)) (95% CI). Two out of nine studies of HOMA-IR were included in the meta-analysis: one by Ibero-Baraibar et al. (2016) and another by Leyva-Soto et al. (2018). They showed a significant reduction in HOMA-IR data in the intervention group [31,35]. Meta-analysis of HOMA-IR indicated substantial heterogeneity among studies, at I^2^ = 70%. The mean HOMA-IR value in the intervention group was reduced ((−0.24 (−0.52 to 0.04)) (95% CI). Eight studies described changes in insulin levels and were included in the meta-analysis. Ibero-Baraibar et al. (2016) and Nickols-Richardson et al. (2014) found a significant decrease in insulin level in the intervention group compared with the control group [31,34]. Meta-analysis of insulin indicated substantial heterogeneity among studies, at I^2^ = 70%. The mean insulin value in the intervention group was reduced ((−0.15 (−0.80 to 0.50)) (95% CI).

There are some limitations in this systematic review and meta-analysis. Firstly, in the literature search, only fifteen studies fulfilled the inclusion criteria, even though we included four databases with 4664 articles in the initial search. Furthermore, most of the studies had a reduced sample size. Therefore, the health benefits due to Mexican ancestral food consumption, such as a reduction in lipid biochemical profile, could not always be observed in the results of the included studies. Additionally, there is only a small overall effect size of nopal, cacao, avocado, and common bean in lowering total cholesterol, triglycerides, LDL-c, and HDL-c showed in this systematic review and meta-analysis. Secondly, a probable limitation observed in the studies included in this meta-analysis are the complications to blind the participants and the researcher; unfortunately, this limitation is common in several nutritional intervention studies, which remains a risk of bias. Thirdly, the amount and presentation of the Mexican ancestral foods supplemented was different in each study; specifically, dark chocolate products are very heterogeneous, containing variable amounts of cocoa solids, reflecting the high heterogeneity between studies in our meta-analyses. Furthermore, the different doses and forms of cocoa, nopal, avocado, and common bean used in the interventions lead to different amount of calories, sugar, fiber, and fat content of the investigated products. Moreover, the duration of intervention of Mexican ancestral foods was different in each study included. Because of these multiple variations, it is probable that no significant improvement in anthropometric outcomes could be observed.

## 5. Conclusions

The presented results showed that supplementation with Mexican ancestral foods significantly improves body mass index in obese patients. This review makes several recommendations for future research, including the need for additional large, high-quality randomized control trials that are sufficiently powered and done internationally with a more diverse mix of participant genders and ages. Feasibility studies should investigate ways to keep participants motivated during interventions and reduce high attrition rates. While the majority of the studies included in this evaluation examined the effectiveness of the interventions in relation to their primary and secondary outcomes, important measures relating to the methods of implementing these dietary interventions were not scrutinized. Thus, more studies are required to determine the ideal doses, optimal administration or presentation, and duration of intervention. In addition, studies that evaluate a possible synergistic effect of supplementation of a mix of several Mexican traditional foods such as cocoa, nopal, avocado, and common bean are needed.

## Figures and Tables

**Table 1:** Inclusion and exclusion criteria of selected articles.

Criteria	Inclusion	Exclusion
Article type	Research original articles	Systematic review, book chapter, conference proceedings
Study type	Clinical trials and observational studies	Animal and in vitro studies
Participants	Adults aged 18 years and over with obesity or diabetes mellitus type 2 or metabolic syndrome	Children and adolescents
Mexican ancestral food	Cacao or nopal or common bean or avocado	Another Mexican ancestral food
Outcomes	Baseline and final data at least one of: BMI, waist circumference, total cholesterol, LDL-c, HDL-c, glucose, HOMA-IR, insulin	Only change percentage or graphics of outcomes
Language	English	Non-English
Period of time	Between 2012 and 2022	Published before 2012

**Table 2:** Characteristics of included studies.

Author (Year of Publication)	Country	Study Design	Sample Size (Considering Dropouts)	Mean Age (SD)	Baseline BMI (SD)	Duration of Intervention	Condition	Intervention Groups	Main Outcomes
West et al. (2013) [36]	United States	Randomized controlled double-blind cross-over trial	30	All subjects: 52.6 (0.40)	All subjects: 27.4 (0.5)	4 weeks	Overweight	Group 1: 3 mg per day of flavonoid in a low-concentration flavonoid chocolate Group 2: 37 g per day of dark chocolate plus 22 g per day of natural cocoa.	No changes in body weight, BMI or waist circumference No variations in glucose and lipid profile
Nickols Richardson et al. (2014) [34]	United States	Randomized controlled double-blind cross-over trial	51	All subjects: 36.0 (1.1)	All subjects: 30.8 (0.9)	18 weeks	Obesity	Group 1: 236 mL unsweetened natural cocoa beverage per day and one 1.45 oz. dark chocolate Group 2: 236 mL of unsweetened vanilla beverage per day and two sweet snacks without chocolate	↓ Body weight, glucose, insulin No modification in lipid profile
Munguía Levy et al. (2015) [32]	México	Randomized controlled double-blind cross-over trial	15	All subjects: 20–60 (not specified)	All subjects: 28.6 (0.9)	4 weeks	Obesity and metabolic syndrome	Group 1: cocoa bean extract powder (80 mg flavonoids) Group 2: placebo powder (no sugar, no flavonoids)	↓ Body weight and waist circumference ↓ Total cholesterol, triglycerides, LDL-c and HDL-c
Ibero-Baraibar Idoia et al. (2015) [30]	Spain	Randomized controlled double-blind cross-over trial	23	Control: 57 (5.0) Intervention: 58 (5.6)	Control: 30.3 (1.9) Intervention: 30.7 (2.5)	4 weeks	Obesity	Group 1: diet restricted in 15% of energy + prepared meals + 1.4 g of cocoa extract (645 mg of polyphenols) Group 2: 15% energy-restricted diet + prepared foods	↓ Body weight in both groups No change in neither insulin level nor fasting glucose in both groups ↓ Total cholesterol, triglycerides, LDL-c, HDL-c in both groups
Ibero-Baraibar Idoia et al. (2016) [31]	Spain	Randomized controlled double-blind trial	22	Control: 57 (4.9) Intervention: 59 (5.4)	Control: 30.2 (2.2) Intervention: 31.4 (2.6)	28 days	Obesity	Group 1: meal without the cocoa extract daily. Group 2: meal enriched with 1.4 g/day of cocoa extract (415 mg flavanols).	No changes in BMI, glucose, insulin, total cholesterol, and HDL-c
Pignotti et al. (2016) [43]	United States	Randomized, crossover single-blinded trial	16	All subjects: 46.5 (13.9)	Control: 31.5 (5.9) Intervention: 31.4 (5.8)	2 weeks	Obesity with moderate hypercholesterolemia	Group 1: 1 cup of peeled cucumbers (140 g) with each of their two main meals per day. Group 2: 1 cup of nopal (140 g) with each of their two main meals per day.	No changes in BMI, waist circumference, glucose, insulin, total cholesterol, triglycerides, LDL-c, and HDL-c
Dicks et al. (2018) [29]	Germany	Randomized, placebo-controlled, double-blinded trial	35	Control: 62.8 (1.6) Intervention: 65.6 (2.6)	Control: 29.3 (26.0; 33.8) # Intervention: 30.2 (26.5; 34.7) #	12 weeks	Type 2 diabetes mellitus and Obesity	Group 1: capsules of cocoa powder (2.5 g/day) Group 2: capsules of pure microcrystalline cellulose (2.5 g/day)	No changes in glucose, insulin, HOMA-IR, triglycerides, total cholesterol, LDL-c, and HDL-c
Leyva-Soto et al. (2018) [35]	Mexico United States	Randomized controlled double-blind cross-over trial	84	All subjects: 23.8 (3.4)	Control: 32.1 (3.8) Intervention: 31.4 (3.2)	6 months	Obesity	Group 1: 2 g dark chocolate (70% cocoa) daily Group 2: 2 g milk chocolate daily	↓ Total cholesterol, triglycerides, LDL-c ↓ Waist circumference ↓ HOMA-IR
Munguía-Levy et al. (2019) [33]	Mexico	Randomized, double-blinded placebo-controlled	40	Initial 60.4 (3.2) 63 (3.3) Follow-up 63.6 (2.4)	Not specified	12 weeks 8 weeks	Overweight/ Obesity	Group 1: cocoa-free skim milk-based powder beverage (containing colorants and flavors) once a day Group 2: flavonoid-free alkalinized natural cocoa powder (0 mg of flavonoids) once a day. Group 3: flavonoid-rich natural cocoa powder (179 mg of flavonoids), once a day	↓ Glucose, total cholesterol and triglycerides, LDL-c, and HDL-c.
Wang et al. (2020) [38]	China	Randomized, double-blinded placebo-controlled	120	Control: 42.9 (8.0) Intervention: 42.4 (8.5)	Control:28.87 (0.83) Intervention:28.97 (0.90)	35 days	Overweight/ Obesity	Group 1: capsules of maltodextrin (2400 mg) before 3 daily meals Group 2: capsules of common bean extract (2400 mg) before 3 daily meals	↓ Body weight, body mass index, fat mass, adipose tissue thickness, and waist circumference
Khan et al. (2021) [39]	United States	Randomized controlled double-blind cross-over trial	37	34.5 (5.9)	Control: 33(6.2) Intervention: 32.1 (6.0)	12 weeks	Obesity	Group 1: daily meal with 1 fresh Hass avocado Group 2: isocaloric meal using similar ingredients without avocado	↓ Visceral adipose tissue No modifications in insulin and HOMA-IR
Escobedo et al. (2021) [37]	Mexico	Randomized controlled double-blind cross-over trial	20	26.0 (4.9)	27.2 (1.2)	4 weeks	Overweight	Group 1: usual diet (no placebo) Group 2: usual diet + common bean snack (32 g common bean/day)	↓ Apolipoprotein B No modifications in total cholesterol and triglycerides, LDL-c, and HDL-c.
Corona-Cervantes et al. (2022) [42]	México	Prospective study	36	22.1 (2.6)	Control: 21.5 (1.9) Intervention: 35.1 (4.5)	30 days	Obesity	Group 1: 2100 g nopal per week. Group 2: 2100 g nopal per week and 30 min of light walking per day.	↓ Body weight and waist circumference ↓ Glucose, total cholesterol, and HDL-c
Lichtenstein et al. (2022) [40]	United States	Randomized controlled, parallel-arm, unblinded study	1008	50.3 (14.0)	Control: 32.9 (5.3) Intervention: 33.2 (5.6)	6 months	Metabolic Syndrome with elevated waist circumference.	Group 1: One avocado per day, without changes in eating habits, usual diet and lifestyle. Group 2: Avocado intake to ≤2 avocados/month, without changes in eating habits, usual diet and lifestyle.	↓ Total cholesterol and LDL-c No changes in body mass index, glucose, HDL-c, and insulin
Zhang et al. (2022) [41]	United States	Randomized controlled, two-arm, parallel trial	93	Control: 42.7 (12.5) Intervention: 40.6 (11.8)	Control: 32.8 (3.88) Intervention: 32.3 (3.90)	12 weeks	Obesity and Insulin resistance	Group 1: One avocado per day, (48–168 g pulp) Group 2: Low-fat/low-fiber/high-carbohydrate food (mini bagels, pierogis, fruit juice, waffle, instant oatmeal).	No changes in body mass index, total cholesterol, triglyceride, glucose, HDL-c, and LDL ↓ Fasting insulin, HbA1c

BMI, Body Mass Index; T2DM, Type 2 Diabetes Mellitus; LDL-c, low density lipoprotein cholesterol; HDL-c, high density lipoprotein cholesterol; HbA1c, hemoglobin A1c; HOMA-IR, homeostatic model assessment for insulin resistance. ↓ diminution in control variables; # logarithmized data used for statistical tests.

## Data Availability

Data is contained within the article.

## Supplementary Materials

The following supporting information can be downloaded at: https://www.mdpi.com/article/10.3390/foods12061177/s1, PICOS criteria for database inclusion and exclusion of studies, search terms, and strategy research.
