# Supplementary material for: Mexican Ancestral Foods (Theobroma cacao, Opuntia ficus indica, Persea americana and Phaseolus vulgaris) Supplementation on Anthropometric, Lipid and Glycemic Control Variables in Obese Patients: A Systematic Review and Meta-Analysis
Source: Foods. 2023 Mar 10;12(6):1177. doi: 10.3390/foods12061177 (PMC10047948; doi:10.3390/foods12061177)
Supplement: Supplementary file 1 [file foods-12-01177-s001.zip › foods-2220529-supplementary.pdf]

Research question: Does ancestral food supplementation improve lipid profile, glycemic control or reduce body mass index in patients with overweight or obesity?

PICOS criteria for inclusion and exclusion of studies

Parameter	Criteria
**Population**	**Inclusion criteria:**<br>1) Randomized controlled trials and observational studies<br>2) Clinical trials and observational studies involving adults with obesity or patients with obesity and diabetes mellitus type 2 or metabolic syndrome.<br>3) Studies that assess the effect of ancestral Mexican food (nopal, cacao, common bean or avocado) in at least one intervention arm.<br>**Exclusion criteria:**<br>1) Animal model or *in vitro* studies.<br>2) Studies in patients with diseases other than obesity, diabetes mellitus type 2 or metabolic syndrome.<br>3) Studies in which ancestral Mexican food is combined with another supplement in the same supplementation.<br>4) Studies with children or adolescent<br>5) Meetings or conferences abstracts or reviews<br>6) Clinical trials registries
**Intervention**	Cacao or nopal or common bean or avocado supplementation
**Comparator**	Placebo or Control group, no intervention or other intervention different from ancestral Mexican food
**Outcomes**	**Primary outcomes:** body weight, body mass index, waist circumference, total cholesterol, triglycerides, LDL-cholesterol and HDL-cholesterol.

	**Secondary outcomes:** fasting glucose, 2-hour plasma glucose or glucose tolerance test, insulin levels and HOMA-IR.
**Study design**	Randomized controlled trials and observational studies

Search terms

- **Ancestral Mexican supplementation**: cacao OR cocoa OR dark chocolate OR Theobroma cocoa OR nopal OR Opuntia ficus indica OR Prickly pear cactus OR common bean OR Phaseolus vulgaris OR avocado OR Persea americana
- **Obesity:** obesity OR weight loss OR weight reduce OR weight decrease OR obese OR central obesity OR overweight OR adipose tissue OR fat mass OR adiposity OR waist circumference 0R body mass index OR body mass index (BMI)
- **Cholesterol:** blood cholesterol OR cholesterol esters OR Hypercholesterolemia
- **Triglycerides**: blood triglycerides OR hypertriglyceridemic waist OR triacylglycerol OR
- triacylglycerol OR triglycerides
- **Fasting glucose:** blood Sugar OR blood glucose OR fasting glucose OR fasting plasma glucose OR impaired fasting glucose
- **Insulin:** insulin resistance OR HOMA-IR OR homeostasis model assessment.
- All reference lists of eligible articles were hand-searched to avoid omitting any pertinent articles. In addition, unpublished articles and grey literature such as conference papers, thesis, and patents were not included in this study.

PUBMED

1. ((((((((((((((((((((((((“Obesity”[Title/Abstract]) OR (“Obese”[Title/Abstract])) OR ("weight loss"[Title/Abstract])) OR ("weight reduce"[Title/Abstract])) OR ("weight decrease"[Title/Abstract])) OR (“central obesity”[Title/Abstract])) OR ("overweight" [Title/Abstract])) OR ("adipose tissue"[Title/Abstract])) OR ("fat mass" [Title/Abstract])) OR ("adiposity"[Title/Abstract])) OR (“body mass index” [Title/Abstract])) OR (“body mass index (BMI)” [Title/Abstract])) OR (“body fat” [Title/Abstract])) OR (“waist circumference” [Title/Abstract])) AND (Cacao[Title/Abstract])) OR (Cocoa[Title/Abstract])) OR (Dark chocolate[Title/Abstract])) OR (Theobroma cacao[Title/Abstract])) OR (Nopal[Title/Abstract])) OR (Opuntia ficus indica[Title/Abstract])) OR (Prickly pear cactus [Title/Abstract])) OR (Common bean[Title/Abstract])) OR (Phaseolus vulgaris[Title/Abstract])) OR (Avocado[Title/Abstract])) OR (Persea americana[Title/Abstract])

2. (((((((((((((((((Prediabetes[Title/Abstract])) OR (Prediabetic[Title/Abstract])) OR ("Prediabetic State"[Title/Abstract])) OR ("Diabetes Mellitus"[Title/Abstract])) OR ("Type 2 Diabetes"[Title/Abstract])) OR ("Type 2 Diabetes mellitus"[Title/Abstract])) OR (Diabetes[Title/Abstract])) OR ("Type II Diabetes Mellitus"[Title/Abstract])) AND (Cacao[Title/Abstract] OR (Cocoa[Title/Abstract])) OR (Dark chocolate[Title/Abstract])) OR (Theobroma cacao[Title/Abstract])) OR (Nopal[Title/Abstract])) OR (Opuntia ficus indica[Title/Abstract])) OR (Prickly pear cactus [Title/Abstract])) OR (Common bean[Title/Abstract])) OR (Phaseolus vulgaris[Title/Abstract])) OR (Avocado[Title/Abstract])) OR (Persea americana[Title/Abstract])

3. ("Blood Sugar"[Title/Abstract] OR "Blood glucose"[Title/Abstract] OR "Fasting plasma glucose"[Title/Abstract] OR "Impaired fasting glucose"[Title/Abstract] OR "Insulin Resistance"[Title/Abstract] OR "HOMA-IR"[Title/Abstract] OR "HOMA IR"[Title/Abstract] OR "Homeostasis model assessment" [Title/Abstract] OR "Oral glucose tolerance test" [Title/Abstract] OR "Glucose tolerance test" [Title/Abstract] OR OGTT[Title/Abstract] OR "Glucose intolerance" [Title/Abstract] OR "Blood cholesterol"[Title/Abstract] OR "Cholesterol esters"[Title/Abstract] OR "Hypercholesterolemia"[Title/Abstract] OR "Blood triglycerides"[Title/Abstract] OR "Hypertriglyceridemic waist"[Title/Abstract] OR "Triacylglycerol"[Title/Abstract] OR "LDL-cholesterol"[Title/Abstract] OR “Low density lipoprotein" [Title/Abstract] OR “HDL-cholesterol" [Title/Abstract] OR "High density lipoprotein cholesterol" [Title/Abstract] AND (Cacao[Title/Abstract] OR Cocoa[Title/Abstract]) OR Dark

chocolate[Title/Abstract] OR Theobroma cacao[Title/Abstract] OR Nopal[Title/Abstract] OR Opuntia ficus indica[Title/Abstract] OR Prickly pear cactus [Title/Abstract] OR Common bean[Title/Abstract] OR Phaseolus vulgaris[Title/Abstract] OR Avocado[Title/Abstract] OR Persea americana[Title/Abstract])

Filters: article type: clinical trial and observational study, publication date: January 01, 2012 to December 31, 2022 and language: English.

Results

1. 280

2. 164

3. 181

Total reports from Pubmed: 625

Cochrane Central Register of Controlled Trials (CENTRAL)

1. (Cacao)ti,ab,kw, (Cocoa)ti,ab,kw OR (Dark chocolate)ti,ab,kw OR (Theobroma cacao)ti,ab,kw OR (Nopal)ti,ab,kw OR (Opuntia ficus indica)ti,ab,kw OR (Prickly pear cactus) ti,ab,kw OR (Common bean)ti,ab,kw OR (Phaseolus vulgaris)ti,ab,kw OR (Avocado)ti,ab,kw OR (Persea americana) ti,ab,kw

2. (Obesity)ti,ab,kw OR (Obese)ti,ab,kw OR (Weight loss)ti,ab,kw OR (Weight reduce)ti,ab,kw OR (Weight decrease)ti,ab,kw OR (Central obesity)ti,ab,kw OR (Overweight)ti,ab,kw OR (Adipose tissue)ti,ab,kw OR (Fat mass)ti,ab,kw OR (Adiposity)ti,ab,kw OR (Body mass index) ti,ab,kw OR (Body mass index BMI) ti,ab,kw OR (Body fat) ti,ab,kw OR (Waist circumference) ti,ab,kw

3. (Prediabetes)ti,ab,kw OR (Prediabetic)ti,ab,kw OR ("Prediabetic State")ti,ab,kw OR ("Diabetes Mellitus")ti,ab,kw OR ("Type 2 Diabetes")ti,ab,kw OR ("Type 2 Diabetes mellitus")ti,ab,kw OR (Diabetes)ti,ab,kw OR ("Type II Diabetes Mellitus")ti,ab,kw

4. (“Blood Sugar”) ti,ab,kw OR (“Blood Glucose”)ti,ab,kw OR (“Fasting Glucose”)ti,ab,kw OR (“Fasting Plasma Glucose”)ti,ab,kw OR (“Impaired fasting glucose”)it,ab,kw OR (Insulin resistance)ti,ab,kw OR (“HOMA-IR”)ti,ab,kw OR (“HOMA IR”)ti,ab,kw OR (“Homeostasis model assessment”)ti,ab,kw OR ("Blood cholesterol")ti,ab,kw OR ("Cholesterol esters")ti,ab,kw OR ("Hypercholesterolemia") ti,ab,kw OR ("Blood triglycerides")ti,ab,kw OR ("Hypertriglyceridemic waist")ti,ab,kw OR ("Triacylglycerol")ti,ab,kw OR ("LDL-cholesterol")ti,ab,kw OR (“Low density lipoprotein")ti,ab,kw OR (“HDL-cholesterol")ti,ab,kw OR ("high density lipoprotein cholesterol")ti,ab,kw

5. #1 and #2: 19

6. #1 and #3: 10

7. #1 and #4: 5

Filters: publication date from January 2012 to December 2022.

Total reports from Cochrane Central Register of Controlled Trials (CENTRAL): 34

WEB OF SCIENCE

1. ((((((((((TS=(Cacao)) OR TS=(Cocoa)) OR TS= (dark chocolate)) OR TS= (theobroma cacao)) OR TS=(nopal)) OR TS=(opuntia ficus indica)) OR TS=(common bean)) OR TS=(phaseolus vulgaris)) OR TS=(avocado)) OR TS=(persea americana))

2. ((((((((((((((TS=(Obesity)) OR TS=(Obese)) OR TS= (Weight loss)) OR TS= (Weight reduce)) OR TS= (Weight decrease)) OR TS= (Central obesity)) OR TS= (Overweight)) OR TS= (Adipose tissue)) OR TS= (Fat mass)) OR TS= (Adiposity)) OR TS= (Body mass index)) OR TS= (Body mass index BMI)) OR TS= (Body fat)) OR TS= (Waist circumference))

3. ((((((((TS=(Prediabetes)) OR TS=(Prediabetic)) OR TS=(Prediabetic State)) OR TS =(Diabetes Mellitus)) OR TS =(Type 2 Diabetes)) OR TS =(Type 2 Diabetes mellitus)) OR TS =(Diabetes)) OR TS =(Type II Diabetes Mellitus))

4. (((((((((((((((((((TS= (Blood Sugar)) OR TS = (Blood Glucose)) OR TS = (“Fasting Glucose”)) OR TS = (“Fasting Plasma Glucose”)) OR TS=(“Impaired fasting glucose”)) OR TS= Insulin resistance)) OR TS = (“HOMA-IR”)) OR TS = (“HOMA IR”)) OR TS = (“Homeostasis model assessment”)) OR (TS = ("Blood cholesterol")) OR TS = ("Cholesterol esters")) OR TS= ("Hypercholesterolemia")) OR TS = ("Blood triglycerides")) OR TS = ("Hypertriglyceridemic waist")) OR TS = ("Triacylglycerol")) OR TS = ("LDL-cholesterol")) OR TS = (“Low density lipoprotein")) OR TS = (“HDL-cholesterol")) OR TS = ("High density lipoprotein cholesterol"))

Filters: document type: article, year: 2012-2022 and language: English.

5. #1 and #2

Results: 1337

6. #1 AND #3

Results: 312

7. #1 and #4

Results:606

Total reports from Web of Science: 2,255

SCOPUS

1. ((“Cacao” OR “Cocoa” OR “Dark chocolate” OR “Theobroma cacao”)) OR ((“Nopal” OR “Opuntia ficus indica” OR “Prickly pear cactus”)) OR ((“Common bean” OR “Phaseolus vulgaris”)) OR ((“Avocado” OR “Persea americana”))

2. (“Obesity”) OR (“Obese”) OR (“Weight loss”) OR (“Weight reduce”) OR (“Weight decrease”) OR (“Central obesity”) OR (“Overweight”) OR (“Adipose tissue”) OR (“Fat mass”) OR (“Adiposity”) OR (“Body mass index”) OR (“Body mass index BMI”) OR (“Body fat”) OR (“Waist circumference”)

3. (“Prediabetes”) OR (“Prediabetic”) OR (“Prediabetic State”) OR (“Diabetes Mellitus”) OR (“Type 2 Diabetes”) OR (“Type 2 Diabetes mellitus”) OR (“Diabetes”) OR (“Type II Diabetes Mellitus”)

4. ((“Blood Sugar” OR “Blood Glucose”)) OR ((“Fasting Glucose” OR “Fasting Plasma Glucose”)) OR ((“Impaired fasting glucose” OR “Insulin resistance”)) OR ((“HOMA-IR” OR “HOMA IR” OR “Homeostasis model assessment”)) OR (("Blood cholesterol" OR "Cholesterol esters" OR "Hypercholesterolemia")) OR (("Blood triglycerides" OR "Hypertriglyceridemic waist" OR "Triacylglycerol")) OR (("LDL-cholesterol" OR “Low density lipoprotein")) OR ((“HDL-cholesterol" OR "High density lipoprotein cholesterol"))

Filter: search within: article title, abstract, keywords, document type: article, year: 2012-2022 and language: English.

5. #1 and #2

Results: 689

6. #1 AND #3

Results:373

7. #1 and #4

Results:688

Total reports from Scopus: 1750
